# Genetics of aging, health, and survival: dynamic regulation of human longevity related traits

**DOI:** 10.3389/fgene.2015.00122

**Published:** 2015-04-13

**Authors:** Anatoliy I. Yashin, Deqing Wu, Liubov S. Arbeeva, Konstantin G. Arbeev, Alexander M. Kulminski, Igor Akushevich, Mikhail Kovtun, Irina Culminskaya, Eric Stallard, Miaozhu Li, Svetlana V. Ukraintseva

**Affiliations:** ^1^Biodemography of Aging Research Unit, Center for Population Health and Aging, Social Science Research Institute, Duke UniversityDurham, NC, USA; ^2^Integrative Genomic Analysis Shared Resource, Duke Center for Genomic and Computational Biology, Duke UniversityDurham, NC, USA

**Keywords:** longitudinal data, genetics of longevity, genetics of cancer, CVD, gene-environment interaction, smoking and life span, aging changes

## Abstract

**Background:** The roles of genetic factors in human longevity would be better understood if one can use more efficient methods in genetic analyses and investigate pleiotropic effects of genetic variants on aging and health related traits.

**Data and methods:** We used EMMAX software with modified correction for population stratification to perform genome wide association studies (GWAS) of female lifespan from the original FHS cohort. The male data from the original FHS cohort and male and female data combined from the offspring FHS cohort were used to confirm findings. We evaluated pleiotropic effects of selected genetic variants as well as gene-smoking interactions on health and aging related traits. Then we reviewed current knowledge on functional properties of genes related to detected variants.

**Results:** The eight SNPs with genome-wide significant variants were negatively associated with lifespan in both males and females. After additional QC, two of these variants were selected for further analyses of their associations with major diseases (cancer and CHD) and physiological aging changes. Gene-smoking interactions contributed to these effects. Genes closest to detected variants appear to be involved in similar biological processes and health disorders, as those found in other studies of aging and longevity e.g., in cancer and neurodegeneration.

**Conclusions:** The impact of genes on longevity may involve trade-off-like effects on different health traits. Genes that influence lifespan represent various molecular functions but may be involved in similar biological processes and health disorders, which could contribute to genetic heterogeneity of longevity and the lack of replication in genetic association studies.

## Introduction

The literature review on the genetic influence on human aging and longevity indicates limited progress in identifying genetic mechanisms regulating human aging, health, and longevity using genome wide association studies (GWAS). Most associations detected in these studies tend to be weak and have not reached the genome-wide level of statistical significance. They also suffer from the lack of replication in studies of independent populations. What factors and conditions might be responsible for such a situation? What methods and approaches have to be used to properly address these issues?

The literature on genetic studies of human longevity lacks an appropriate biologically-based conceptual framework that would allow one to efficiently address questions about the dynamic roles of genes in forming aging and longevity related traits. Such a framework would help specify necessary steps for efficient comprehensive analyses of corresponding genetic mechanisms using available data. The approaches based on the systems biology of aging, health, and longevity (Kriete, 2013; Yashin and Jazwinski, 2014) merged with biodemography of aging methods (Arbeev et al., 2011; Yashin et al., 2012b) and statistical modeling (Yashin et al., 2012a; Arbeev et al., 2014) have the potential to facilitate progress in this direction.

The literature review also revealed the underutilized research potential of available data that were used in genetic analyses of aging and longevity traits. The possibility of more efficient analyses of genetic associations with such traits was demonstrated in Yashin et al. (1999, 2000) where the joint analyses of genetic and demographic data in centenarians studies have been performed. The methods of such analyses were further developed and adjusted for the biological and biodemographic nature of the traits and successfully used in data analyses (Yashin et al., 2007, 2013; Arbeev et al., 2011). The results of these analyses indicate that the use of biodemographic ideas, models, and methods could shed more light on genetic mechanisms linking human aging, health, and longevity.

In genetic analyses performed in this paper we used a modified statistical procedure of testing genetic associations with longevity related traits. The populations whose data are used in GWAS often have genetic structures called “population stratification” (PS). Such structures are caused by non-random mating between groups of individuals followed by different patterns of genetic drift of allele frequencies in each group. Without proper controlling for PS in the statistical procedures used in GWAS of complex traits the analyses may result in erroneous (false-positive) associations. To avoid such errors the genetic association studies of complex traits include control for potential PS. The method of principal components analyses (PCA) suggested in Price et al. (2006) identifies several top principal components (PCs) in genetic data and uses them as observed covariates in the statistical estimation procedure in GWAS. Although this approach may be efficient in GWAS of many complex traits it may create problems in genetic association studies of human longevity. This is because the process of mortality selection that takes place in genetically heterogeneous cohorts may generate additional genetic structures in the study populations. This structure which involves genetic variants affecting life span may be captured by the selected principal components. As a result, controlling for PS in GWAS of human aging and longevity may reduce the estimates of associations of genetic variants with longevity traits, i.e., it may substantially attenuate the signals from the genetic variants one wants to detect. In this paper we applied the new procedure for controlling for population stratification (see Section S1 in Supplementary Materials) in GWAS of human life span using data on females from the original cohort of the Framingham Heart Study (FHS). This procedure as well as the results of analyses using traditional and modified methods of controlling for potential PS in GWAS of human longevity are described in Yashin et al. (2014). We investigated the associations of the detected genetic variants with life span in males from the original and in males and females from the offspring FHS cohorts as well as with health related traits and age trajectories of biomarkers from the original cohort. We also investigated functional properties of genes related to detected variants.

## Data and methods

In the discovery phase of the analyses we used the life span data on genotyped females collected in the original cohort of the Framingham Heart Study (FHS) (Giroux, 2013) as well as genetic data. The genetic data were represented by 550,000 SNPs. Genotyping was conducted using Affymetrix 500 K and 50 K (non-overlapping) arrays. The lifespan data were available for 1529 participants from the original FHS cohort. The quality control (QC) procedure included 95% call rate for the sample and 95% call rate for SNPs, and HWE *p*-value > 1E-7. After applying the QC procedure, the data on 1111 individuals with information about lifespan and 429,783 SNPs were available for the analysis. Life spans for 204 study subjects (52 males and 152 females) were censored. We used EMMAX software to perform GWAS of female lifespan data from the original FHS cohort with modified correction for population stratification. In the confirmation phase we used genetic and the life span data on males from the same FHS cohort and the life span data on males and females combined from the offspring FHS cohort. Using available data on cancer (all sites but skin) and CVD as well as longitudinal data on physiological indices we estimated age trajectories of probabilities of staying free of the corresponding diseases for carriers and non-carriers of selected genetic variants as well as associations of these variants with age trajectories of physiological indices. We also investigated how smoking modulates estimated associations. More details about methods of data analyses used in this paper are given in Section S1 of Supplementary Materials.

## Results

### Genetic variants showing association with life span in females are replicated in males

#### Discovery phase

The most significant genetic variants (minor alleles) resulted from GWAS on human life span using female data from the original FHS cohort are shown in Table S1 (in Supplementary Materials) together with corresponding estimates of the effect sizes, *p*-values, genetic frequencies, and other important characteristics of these SNPs.

#### Confirmation phase

To test whether the selected genetic variants also show significant associations with lifespan in males we performed genetic analysis of the ten selected SNPs using lifespan data on 432 males from the original FHS cohort. The same (additive) genetic model and the same observed covariates were used in these analyses. The results are summarized in Table S2 together with corresponding estimates of the effect sizes, *p*-values, genetic frequencies, and other important characteristics of these SNPs. This table shows that the eight genetic variants that had negative associations with lifespan in females also showed statistically significant negative associations with lifespan in males.

Statistical estimates in Tables S1, S2 are obtained using mixed effect regression model with imputed life span data on 204 study subjects). It is unclear whether detected associations remain statistically significant when the methods free of life span imputation are used in the association analyses. To address this issue we evaluated genetic associations of selected genetic variants with mortality risk using Cox's regression model that does not require data imputation. The results of these analyses (Table S3) showed highly significant associations between selected genetic variants and mortality risks.

To test whether detected variants are associated with life spans of the members of the offspring FHS cohort we performed genetic analyses of data for this cohort. Since the life span data for some members of this cohort are censored these analyses have been performed using the Cox's regression model using gender as an observed covariate. All selected variants showed significant associations with mortality risk using data from the offspring cohort (Table S7).

### Empirical survival functions estimated from longitudinal data confirm detected associations

An important advantage of longitudinal data for genetic analyses of lifespan is the opportunity to *verify* the research findings obtaned using regression models in GWAS by constructing and comparing *empirical survival functions* for carriers and non-carriers of selected variants. Note that the case-control analysis, typicaly used in genetic studies of centenarians, does not have data to make such a verification. Since associations of the variants with lifespan in our GWAS have the same direction of the effect in both genders, the Kaplan–Meier estimates of conditional survival functions for the genotyped members of the original FHS cohort were evaluated for males and females combined. In such evaluation we took into account that the life span data on the members of the original FHS cohort are right censored and left truncated. Note that for genetic analyses one should use age at blood collection (not the age at baseline) as the left truncation variable. We calculated these variables using data from the DNA Draw Date file available for FHS data from the dbGaP. Then we calculated ages at death/censoring of those 1111 individuals using age at the first exam and the number of days since the first exam until the death event or censoring which are provided in the Framingham data. Using these data and the R package “survival” we calculated Kaplan–Meier estimates of conditional survival functions for carriers and non-carriers of selected genetic variants who survived until specific ages (e.g., 70 or 80 years).

**Figure 1:**
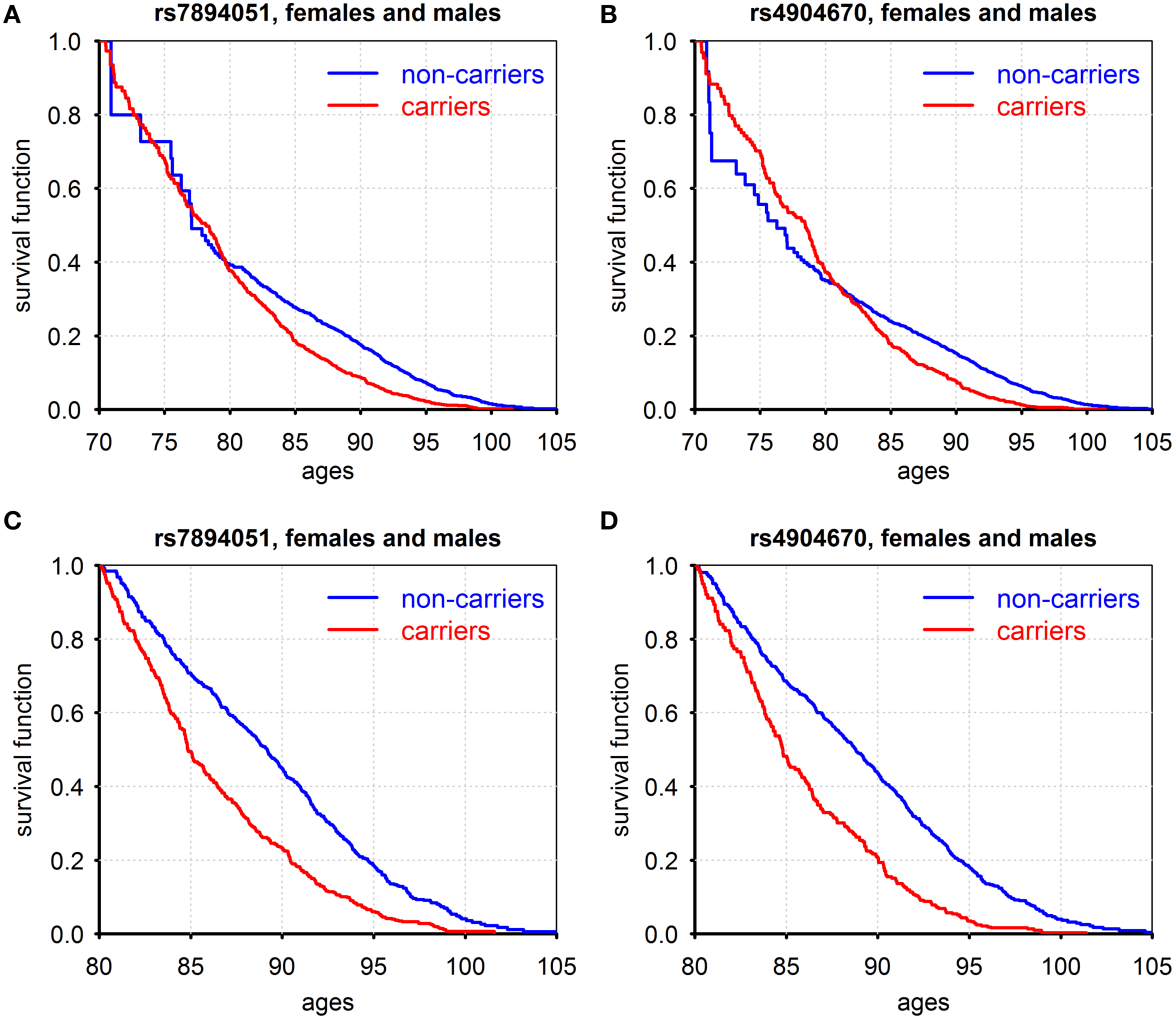
**Kaplan–Meier estimates of conditional survival functions for carriers/non-carriers of minor alleles of rs7894051 (A,C) and rs4904670 (B,D) SNPs surviving to age 70 years (A,B) and age 80 years (C,D)**. Source: Framingham Heart Study, original cohort, genotyped individuals after quality control, females, and males combined.

Figure 1 shows patterns of survival for carriers and non-carriers of the minor allele, for two SNPs: rs7894051 (in ECHS1 gene), and rs4904670 (in NRDE2 gene) that were selected for further analyses after additional QC procedure described in Supplementary Materials.

Figures 1A,B show such functions conditional on survival to age 70 years and the Figures 1C,D show these functions for those who survived age 80 years (for males and females combined). For rs7894051, the negative effect of the minor allele on survival became pronounced only after age 80 years. The probabilities of survival for carriers and non-carriers of these variants at the age interval between 70 and 80 years are about the same. For rs4904670, the negative effect on survival starts from the age 80. This SNP manifests a survival trade-off, with minor allele having positive effect on survival at ages before 80 and negative effect afterwards. This illustrates the changing age-specific influence of genes on vulnerability to death. The role of age as a modulator of genetic effects on lifespan was suggested and discussed in our prior works (De Benedictis et al., 1998; Yashin et al., 1999, 2000, 2001; Ukraintseva, 2005). Later, it was also noted in Atzmon et al. (2006). Age-dependence of genetic effects on health and related traits was also extensively discussed and analyzed in the past by different research groups (e.g., Jarvik et al., 1997) including our own (e.g., Kulminski et al., 2013). Note that the harmful influence of the minor alleles on survival after the age 80 was manifested for all eight genetic variants listed in Table S2. The Cox regression analyses applied to the life span data from the original FHS cohort showed that the difference in survival curves among minor allele carriers and non-carriers for each of the eight SNPs, males and females combined, was highly statistically significant (Table S3 Effects of gene-environment interaction on survival: the case of smoking).

**Figure 2:**
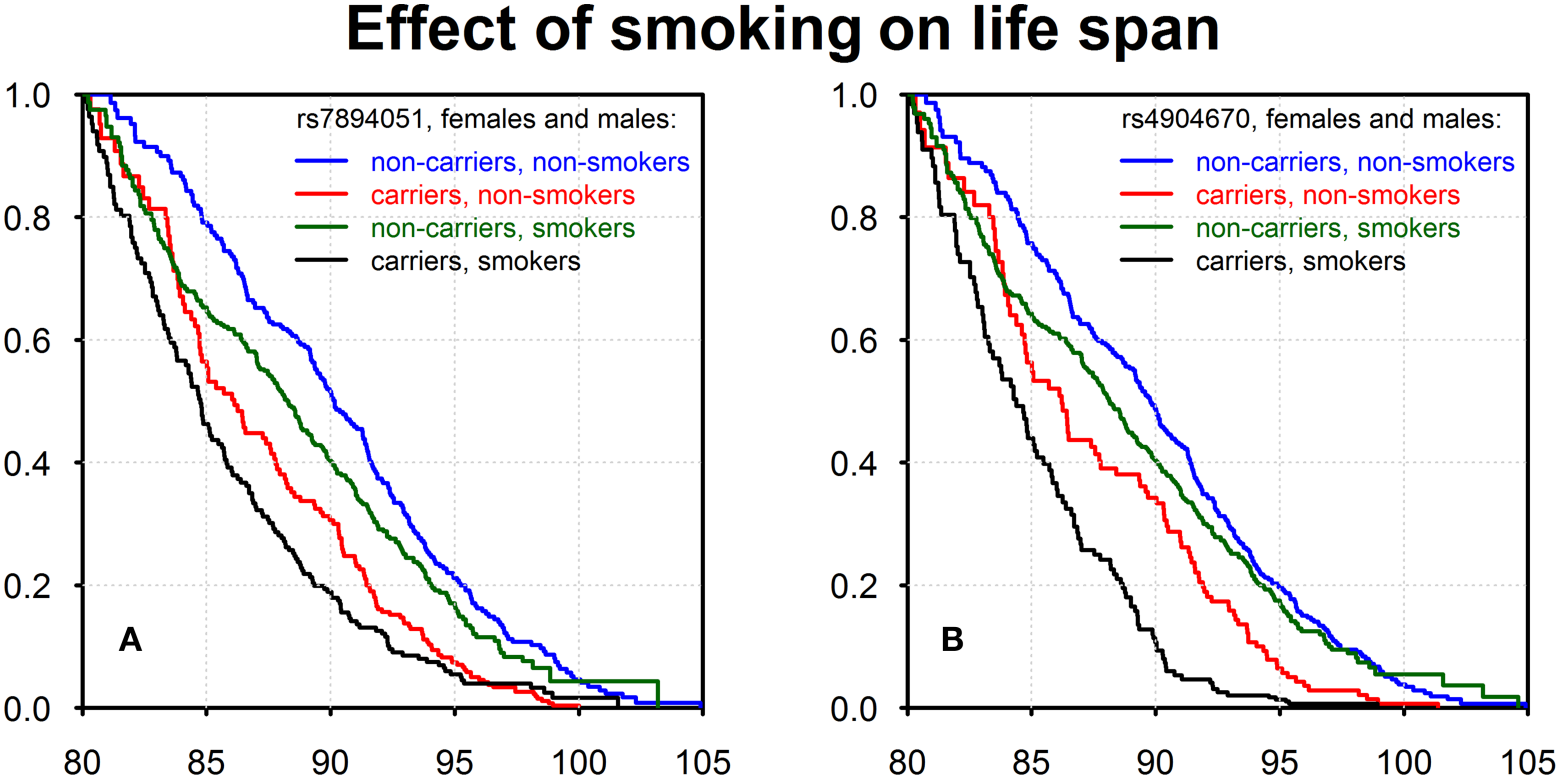
**Kaplan–Meier estimates of survival functions for smokers-carriers, non-smokers-carriers, smokers-non-carriers, and non-smokers-non-carriers of the minor alleles of rs7894051 (A) and rs4904670 (B) SNPs**. Source: Framingham Heart Study, original cohort, genotyped individuals after quality control, females, and males combined.

Using data on the smoking habit (ever or never) as well as life span data on genotyped members of the original FHS cohort (males and females combined) we evaluated effects of interactions of detected genetic variants with the smoking habit on survival. The results are shown in Figure 2.

Figures 2A,B show four survival functions for smoker-carriers, smoker-non-carriers, non-smoker-carriers, and non-smoker-non-carriers of the minor allele, for the two SNPs, rs7894051 and rs4904670, introduced above. These figures show that non-smokers-non-carriers have the best survival, while smokers-carriers have the worst survival among all four groups. This is an important illustration of gene-environment interaction effects on survival, though one can see that the effect of genotype is stronger than the effect of smoking in case of these two SNPs.

### How do detected genetic variants influence incidence rates of cancer and CVD?

**Figure 3:**
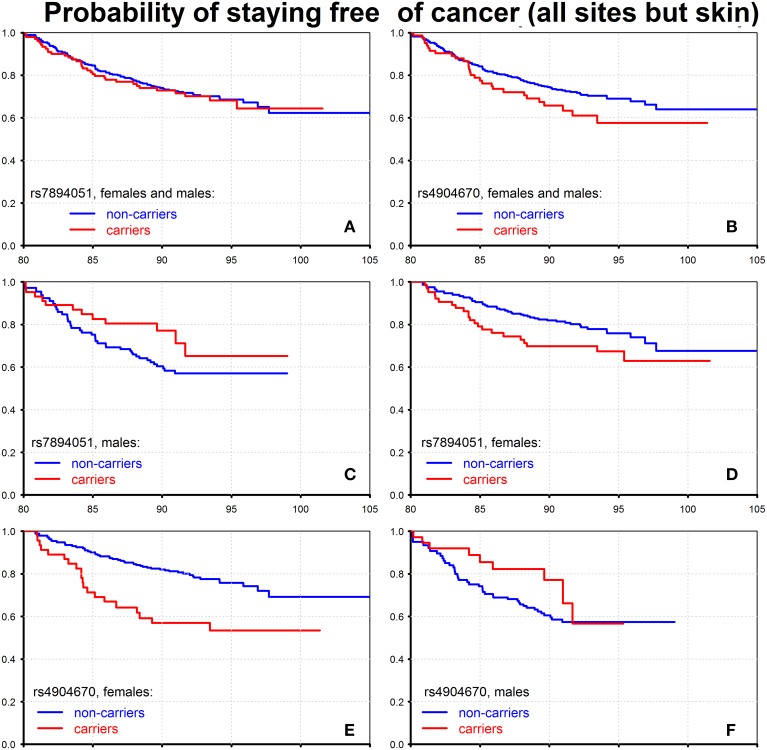
**Effect of individual genetic variants on probability of staying free of cancer of all sites but skin**. The figure shows the Kaplan–Meier estimates of conditional survival functions for individuals who survived to age 80 years: carriers and non-carriers of the minor allele of rs7894051, females and males **(A)**, males **(C)**, females **(D)** and carriers and non-carriers of the minor allele of rs4904670, females and males **(B)**, females **(E)**, males **(F)**. Source: Framingham Heart Study, original cohort, genotyped individuals after quality control.

Using available data on ages at disease onset, we evaluated conditional probabilities of staying free of cancer (all sites but skin), for males and females combined who survived to 80 years of age. The results for the two SNPs, rs7894051 and rs4904670, are shown on Figure 3.

One can see from this figure that when males and females are combined, the rs7894051 does not influence cancer risk (Figure 3A), while carrying the minor allele of rs4904670 (Figure 3B) increases that risk. The pattern observed in Figure 3A may take place when the effect of this allele on cancer risk is opposite in males and females. To test this possibility, we evaluated the age patterns of probabilities of staying free of cancer for males and females separately. The graphs shown in Figures 3C,D provide evidence that such situation does take place: the effects of minor alleles on cancer risk after age 80 were opposite in males and females for both rs7894051 and rs4904670. That is, the presence of the minor allele in person's genome increased the risk of cancer in females and reduced it in males for both the SNPs (Figures 3C–F). However, for rs4904670 the reduction of cancer risk in males did not fully compensate for the increase in such risk in females, so that the total effect of the minor allele of this SNP on cancer risk for males and females combined was detrimental. The effects of rs7894051 and rs4904670 on mortality rates from CVD and cancer are shown in Tables S4, S5 in Supplementary Materials. The vulnerability variants of these two SNPs increased the risk of CHD onset after age 80 (Figures S2, S3 and Table S6). Since carrying the vulnerability allele reduced the cancer risk in males at the same ages, these results suggest potentially important role of antagonistic pleiotropic effects of genes in determining their impact on longevity.

### Longevity related variants can modulate patterns of aging changes in biomarkers

**Figure 4:**
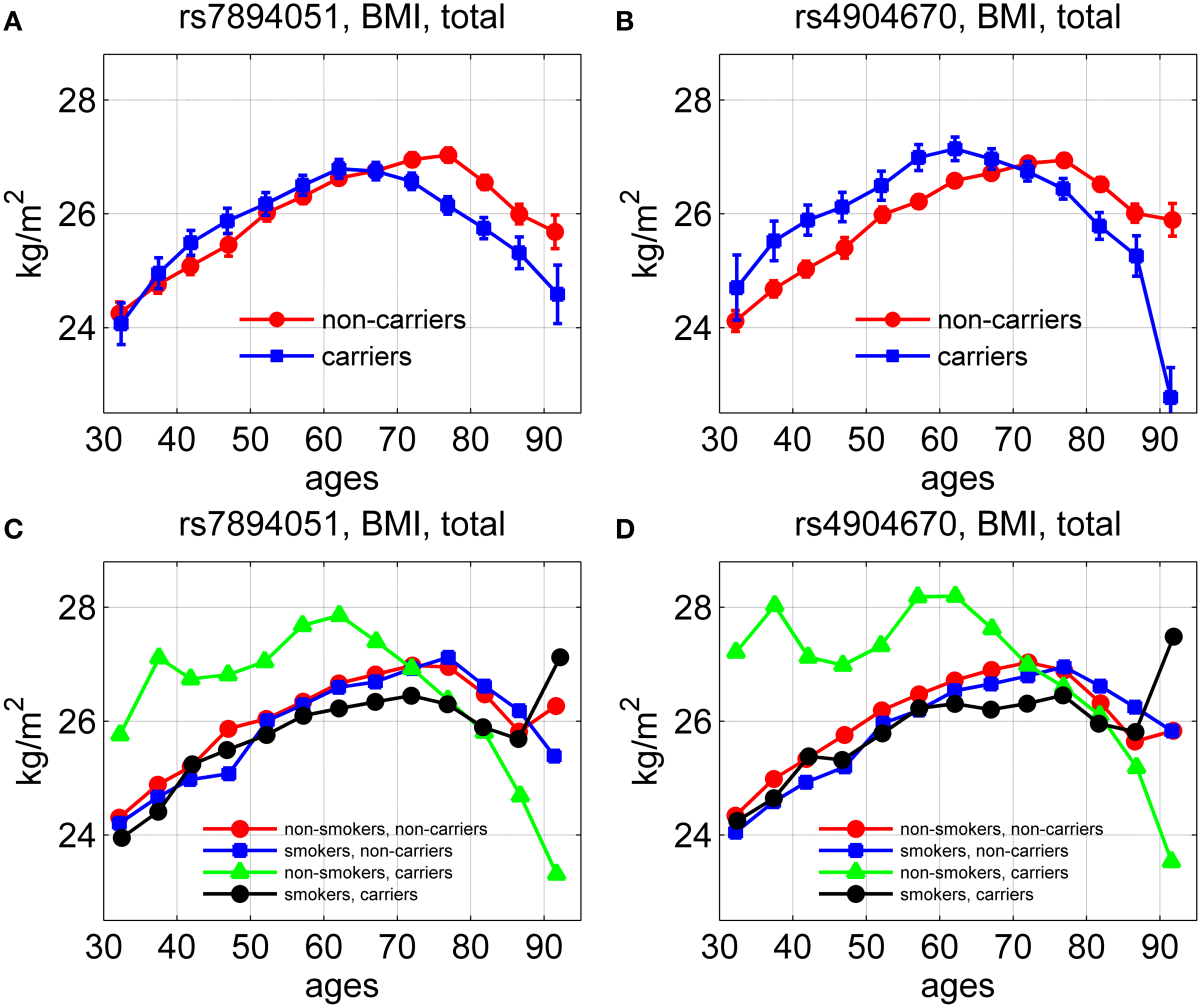
**Average age trajectories of BMI for groups stratified by smoking status and genetic background**. The figure shows the average age trajectories for carriers and non-carriers of the minor alleles of rs7894051 **(A)** and rs4904670 **(B)** SNPs. **(C,D)** show the average age trajectories of BMI for carriers and non-carriers of the same SNPs with different smoking habits.

Using longitudinal data on body mass index (BMI) for genotyped individuals from the original FHS cohort, we evaluated the average age trajectories of BMI for carriers and non-carriers of the minor allele for the same two SNPs, rs7894051 and rs4904670, in males and females. The results are shown in Figure 4.

One can see from Figures 4A,B that between ages 40 and 65 years the average values of BMI for carriers of minor alleles exceeded those for non-carriers, in males and females combined. After this age the curves intersected so that the BMI of the carriers became lower than that of the non-carriers. Figures 4C,D show the effect of smoking habit on the average age trajectories of BMI for rs7894051 and rs4904670 minor-allele carriers and non-carriers. One can see that the average age trajectories for non-smoker-carriers of the minor allele of any of the two SNPs differed from other groups trajectories. It is also seen that smoking did not have a strong influence on the BMI age trajectories for non-carriers, for any of the two SNPs. For rs7894051, values of BMI for non-smoker-carriers were highest at the age interval between 50 and 70 years. For rs4904670 the age trajectory of BMI for the non-smoker-carriers sharply declined after age 70, as for the rs7894051.

### Genes related to the vulnerability variants: biological meaning

Table 1 shows essential characteristics and biological and health effects of the two SNPs, rs7894051 and rs4904670 that passed additional QC we applied as described in Supplement, as well as related genes. We also included rs1794108 to this table and relevant discussion because it is next top SNP by the size of its effect on lifespan (though not by *P*-value), which could aid biological interpretation of the findings. We conducted review of current knowledge about biological and health effects of these SNPs and related genes using up-to-date publications and reliable online sources such as NCBI's PubMed, Entrez Gene, dbSNP, OMIM, Ensembl, GeneCards, GO, and MetaCore from Thomson Reuters. Genes related to SNPs identified in this study (or to SNPs that are in LD with them) are largely involved in mitochondrial oxidation, apoptosis, and protein degradation in cell. These processes are important part of cell/tissue responses to stress or damage. For example, the NRDE2 (C14orf102) gene is linked to PSMC1, a subunit of 26S proteasome involved in protein degradation, essential for cell damage response. PSMC1 may potentially interact with PSMD13, which is another subunit of the same 26S proteasome involved in protein degradation. ECHS1 is involved in mitochondrial oxidation and apoptosis, also essential for cell/tissue response to damage (Table 1).

**Table 1:** **Essential characteristics of the two “vulnerability” SNPs (and their closest genes) associated with reduced lifespan in both sexes in the FHS Original cohort**.

**SNP**	**Ch**	**Gene region**	**Closest gene**	**Gene/protein function in cell and tissue**	**Physiological processes and health disorders associated with the gene/protein**
rs7894051 ^*^This SNP is in LD with rs1049951, exonic non-synonymous SNP in the same gene	10q26.2	Intronic	**ECHS1**—enoyl CoA hydratase, short chain 1, mitoch.	ECHS1 catalyzes the hydration of 2-trans-enoyl-coenzyme A (CoA). Involved in **mitochondrial oxidation** and **apoptosis** suppression (Liu et al., 2010). In LD with 3 intronic SNPs in **PAOX** (oxidase) gene	Overexpressed in different **cancers**; has been implicated in melanoma (Lake et al., 2011), liver and colorectal cancers (Xiao et al., 2013; Zhu et al., 2013; Xie et al., 2014); suggested role in **neurological disorders** (Chiocchetti et al., 2014; Perluigi et al., 2014)
rs4904670	14q32.11	Intronic	**NRDE2** (C14orf102) necessary for RNA interference, domain containing	NRDE2 is highly expressed in the brain (Maiti et al., 2011); linked to **PSMC1**, proteasome 26S subunit, ATPase, 1, involved in **protein degradation**. ^*^The PSMC1 may potentially interact with **PSMD13**, a gene which is in LD with **SIRT3** (see below)	NRDE2 can be associated with **cancer** (melanoma) (Chiu et al., 2014); Suggested role of NRDE2 and PSMC1 in schizophrenia (Maiti et al., 2011); and in **neurodegeneration** (Bedford et al., 2008)
rs1794108	11p15.5	Exon—nonsyn coding	**PSMD13**—proteasome 26S subunit, non-ATPase, 13	PSMD13 is a proteasome regulatory subunit, involved in **protein degradation**. High LD between **PSMD13** and **SIRT3**. ^*^Sirtuins (SIRT1-7) play a central role in epigenetic gene silencing, DNA repair, cell-cycle, microtubule organization, and aging (Giblin et al., 2014)	**Aging, longevity, cancer**. PSMD13-SIRT3 haplotype pools are significantly different between centenarians and younger people (Bellizzi et al., 2007). Plays role in **stem cells aging and stress response** (Brown et al., 2013). SIRT3 can be a tumor promoter or tumor suppressor, depending on context (Alhazzazi et al., 2011)

*Other SNPs are not shown because they did not pass additional QC that we applied as described in Supplement. The SNP rs1794108 was added to this table because it is next top SNP, not by P-value but by the size of its effect on LS. It is also in LD with the SIRT3 gene. The minor allele of this SNP had negative effect on lifespan with significance in males (p < 1.65E-6) and in females (p < 1.91E-4)*.

On the level of health disorders, detected genes were most often involved in cancer and neurological disorders, such as schizophrenia and ASD, which broadly overlaps with biological and health effects of genes found in other studies of aging and longevity (e.g., Walter et al., 2011; Yashin et al., 2012c). The frequent involvement of aging/longevity associated genes in neurological disorders suggests an intriguing possibility that genes predisposing to such disorders may accelerate brain physiological aging, and through this negatively impact longevity (in addition to direct pathological effects). A number of recent studies indicate that this might be the case, especially for schizophrenia (Kochunov et al., 2013; Koutsouleris et al., 2014; Shivakumar et al., 2014; Wright et al., 2014; Silver and Bilker, 2015). Potential mechanisms by which the same genetic factor may influence aging and brain pathology could involve, for example, declines in regenerative response to damage, neural repair, axons outgrowth, and synaptic transmission, typical of both brain aging and brain disorders (Balu and Coyle, 2011; Edwards et al., 2011; Ferguson and Son, 2011; Haroutunian et al., 2014).

As for connection between lifespan related genes and cancer, it appears to be complex and involve trade-offs. Our analyses showed that carrying minor (vulnerability) allele of the two selected SNPs increased cancer risk in females. For males, however, the effect of these variants on cancer was the opposite, and carrying the vulnerability allele reduced the cancer risk in males.

Since most centenarians avoid cancer (Andersen et al., 2005; Joseph et al., 2014), it may seem logical that pro-longevity variants could indeed be among those protecting against cancer. Our results for females are in line with this suggestion. However, vulnerability variants appear to be protective against cancer in males in this study.

This raises another intriguing possibility that pro-longevity variants (in non-carriers of vulnerability alleles) may sometimes do both, promote cancer and at the same time be potentially against certain phenotypes of physical senescence. This could happen because cancer (all sites combined) and senescent phenotypes such as physical frailty, and heart failure due to muscle atrophy, have peak manifestations at different ages: cancer risk reaches its maximum typically before oldest old age (<85), while senescence-related causes become major contributors to mortality risk later (at ages 85+) (Ukraintseva and Yashin, 2003a,b; Ukraintseva et al., 2008; Akushevich et al., 2012). If person who carries such a genetic risk factor for cancer nevertheless survives the period of the highest cancer risk (before age 85), s/he may get an advantage from attenuated physical senescence at older ages, so that such genetic variant may contribute to both longevity and cancer (in case of our study, in males only). Since males and females have markedly different body composition and physical manifestation of senescence, genetic factors could affect this manifestation also differently, and be beneficial or deleterious in only one gender.

Although biological and health effects of identified genes, especially their involvement in damage response, and in cancer, overlap with those earlier found in our and others' studies of aging and lifespan associated genes, the exact replication, such as findings of identical SNPs or genes, is less common across different studies.

## Discussion

The process of population aging in the developed part of the world has dramatic consequences for population health and health-care financing, and makes the reduction of the disease burden of the elderly people a high priority research problem. Useful insights into possible strategies of improving the health of the elderly could be produced from studying the roles of genetic factors in connection with aging, health, and longevity. An important implication of the results of such studies could be the possibility of preventing many aging related chronic diseases by slowing down or postponing individual aging processes.

### Positive implications from modest progress of GWAS of human life span

The genetics of human aging and longevity became the subject of intensive analyses during the last decade ranging from studies of candidate genes (Franceschi et al., 2000; De Benedictis et al., 2001; Tan et al., 2001; Christensen et al., 2006; Christensen, 2008; Willcox et al., 2008) to genome-wide association studies (GWAS) (Lunetta et al., 2007; Newman et al., 2010; Yashin et al., 2010; Deelen et al., 2011, 2013; Nebel et al., 2011) that involved hundreds-of-thousands to millions of SNPs. Although the results of numerous GWAS of human aging and life span have modest success and could not clearly describe factors and mechanisms responsible for exceptional longevity, they have important positive implications. These results helped us identify problems that need to be urgently addressed to have further progress in the research field. Why were most of the detected genetic associations with human life span so weak? Why was it so difficult to replicate the research findings using data on independent studies? What are the mechanisms by which genes influence life span? Can one identify mechanisms that link lifespan-affecting genetic variants with health indicators and aging-related changes in biomarkers? How do detected variants interact with other genetic and non-genetic factors to influence life span? To address these questions, efficient approaches capable of studying the dynamic nature of genetic effects on mortality and morbidity risks, as well as on intermediate variables measured in longitudinal data have to be used. The results obtained with the help of one such approach were described in this paper.

### Genetic estimates in GWAS of human life span require proper control for population stratification

We applied a modified procedure for controlling for potential population stratification in GWAS of human life span to detect genetic variants strongly associated with life span in both males and females and used longitudinal data to address questions about the roles of these variants in health and survival outcomes, age trajectories of biomarkers, as well as the effects of interactions with non-genetic factors. Our aim was to make genetic signals stronger, to evaluate genetic connections among aging-related changes, health, and life span, to elucidate possible reasons for the lack of replication, and to develop useful insights into potential mechanisms of life span regulation. The GWAS performed in these analyses used a modified procedure to control for possible population stratification. Such modification was needed because the traditional procedure may interfere with the process of mortality selection. The confirmation of the genome-wide significant findings in a population of opposite sex (males) in the same (original) cohort and in the offspring generation indicates that these findings are likely to be true-positive. It also indicates that GWAS of human longevity with inappropriate controls for population stratification are likely to suffer from the low level of statistical significance (weak signals) and from the lack of replication.

### Additional QC testing

The genetic data from the original FHS cohort were one of the first prepared for genetic analyses using GWAS, and a substantial portion of genotyped cohort members died before the quality of genotyping was substantially improved. Therefore, these data have higher chances of having genotyping error than more recently produced datasets. Taking this into account we performed testing of the quality of genotyping for eight genetic variants detected in this study (Section S2 in Supplementary Materials). As a result of this testing the two of these SNPs rs7894051 (in ECHS1 gene), and rs4904670 (in NRDE2 gene) were selected for further analyses of their associations with major diseases (cancer and CHD) and physiological aging changes. Thus, although detected genetic associations in eight SNPs were confirmed twice (findings on female data were confirmed using data on males from the original FHS cohort, and then using data from the offspring FHS cohort), additional studies with higher quality genetic data are needed to make final conclusions about the involvement of all eight detected genes in regulation of health, aging, and longevity related traits.

### Benefits of biodemographic information for genetic studies of human longevity

Researchers studying the genetics of human aging and longevity tend to underestimate the role of demographic information in genetic analyses of complex traits. Our analyses show that taking this information into account may substantially improve the quality of genetic analyses. Indeed, researchers studying genetics of human life span tacitly assume that population cohorts are genetically heterogeneous with respect to individual susceptibility to death. The aging of individuals in heterogeneous cohorts is accompanied by the process of mortality selection. The genetically vulnerable individuals tend to die first and genetically robust members of the cohort will survive to the old and the oldest-old ages. In Yashin et al. (1999, 2000, 2007, 2013) and Tan et al. (2003), we developed and tested a series of statistical methods for efficient joint analyses of genetic and demographic information in genetic studies of human longevity. The use of such methods allowed us not only to improve estimates of associations between genetic factors and longevity but also to evaluate and compare age patterns of mortality rates for carriers and non-carriers of candidate alleles and genotypes. Such comparisons are not possible using data on genetic frequencies alone.

### Monotonic vs. non-monotonic age patterns of allele frequencies

Complex diseases, such as cancer, CVD, diabetes, and AD, are major contributors to mortality in old age. Genetic variants which increase risks of such diseases were shown to negatively affect survival and/or be less common among the long-lived people in many studies (e.g., Benes et al., 2001; De Benedictis et al., 2001; Lescai et al., 2009; Park et al., 2009, 2010; Nebel et al., 2011; Ruiz et al., 2011; Yashin and Jazwinski, 2014). The results of our analyses are in line with negative effects of detected alleles on survival after age 80 years. Note that such connection between genetic variants and survival is manifested by *monotonically* declining age patterns of the allele frequencies.

Previously we found that the *non-monotonic* age patterns of allele frequencies are also possible (Yashin et al., 1999, 2000). These patterns (e.g., when the variant's frequency first declines, then reaches minimum, and then increases) corresponds to a very important property of some genetic variants where their effects on mortality risk change with increasing age from detrimental to beneficial. In this case the mortality curves for carriers and non-carriers of the corresponding genetic variant intersect. Possible reasons for such intersection are discussed in Yashin et al. (2001) and Atzmon et al. (2006). Such a pattern could be responsible for the seemingly paradoxical presence of “risk alleles” for complex diseases in genomes of long-lived people, sometimes reported in literature (e.g., De Benedictis et al., 1998; Bladbjerg et al., 1999; Bonafe et al., 1999; Yashin et al., 1999, 2001b; Beekman et al., 2010; Freudenberg-Hua et al., 2014). Thus, the results of genetic studies of human longevity confirm the existence of two groups of genetic variants which can be characterized by different age patterns of genetic frequencies.

### Genetic heterogeneity of complex traits

The results of our analyses show that negative associations of selected genetic variants with human life span in females were confirmed in males; they also influence the incidence rates of cancer of all sites combined (but skin) and CVD. One may argue that the health and longevity traits investigated in this study (i.e., life span and two diseases) are too heterogeneous: individual differences in life spans may depend on susceptibility to different chronic conditions and cause specific mortality risks; cancers of distinct sites are often associated with different genetic and non-genetic factors; the term CVD includes many diseases dealing with cardio-vascular problems, each having its own sets of risk factors; etc. Although these concerns are certainly correct, one often does not have much choice: the selection of a trait for analysis is determined by the available data which has to be enough to get reliable conclusions from the analyses. Studying factors affecting aggregated traits (e.g., cancer of all sites, all-cause, mortality risk, etc.) may help us detect regularities and identify mechanisms that are common for many specific phenotypes (cancers of specific sites, mortality risks by cause, etc.) comprising such traits. An important question is how best to analyze heterogeneous traits when you must? How can an understanding that the trait is genetically heterogeneous affect methods and results of analyses?

Despite the fact that the presence of such heterogeneity is recognized by many researchers in the field, there is no agreement concerning the best strategies for dealing with this issue. The recommendation that follows from our experience would be to investigate genetic heterogeneity by using different statistical models of the connections between genetic factors and phenotypic traits, to consider how the results of analyses (e.g., sets of selected genetic variants) differ for different quality control procedures, to perform careful studies of the non-replicating results, keeping in mind that *different genetic mechanisms may be responsible for the same manifestation of a given trait*. The goal of the analyses thus becomes, not just finding the one or more longevity (or vulnerability) alleles/genotypes responsible for the genetic variation of life span in all individuals, but investigating possible alternative mechanisms responsible for developing this trait. These mechanisms may involve different sets of genes in different individuals or in different groups of individuals. The genetic heterogeneity of longevity could be partly responsible for the lack of replication of respective genetic association studies.

### Genetic mechanisms linking aging, health, and longevity

Although exact replication, such as findings of identical SNPs or genes, is not common across genetic association studies of aging and longevity, biological and health effects of identified genes, especially their involvement in similar damage responses, and in similar pathology, often overlap between the different studies. In our case, the involvement of selected genes (Table 1) in cancer and neurological disorders was reported in both our and others' studies of aging and lifespan associated genes (e.g., Walter et al., 2011).

The results of our review of current knowledge also suggest an intriguing possibility that the vulnerability SNPs in genes involved in neurological disorders might also contribute to accelerated brain aging, and through this negatively impact longevity, which idea deserves further investigation. Connections between lifespan related genes and cancer may involve complex trade-offs. More detail on potential biological mechanisms are provided in section on Biological Meaning above.

In sum, our study demonstrated genetic connections among lifespan, physiological aging changes, and complex diseases. Comparison of functional properties of genes found in this and other genetic studies of aging/longevity indicates that human lifespan may be regulated by different sets of genes; however, these genes tend to be involved in similar biological processes and complex pathology. The fact that the same biological process or health outcome may be realized through the different genes suggests that genes may potentially be interchangeable in their influence on longevity. This could contribute to both genetic heterogeneity of longevity and to a lack of replication in genetic association studies of lifespan and related phenotypes. Additional studies with a higher quality genetic data are needed to support our conclusions.

### Conflict of interest statement

The authors declare that the research was conducted in the absence of any commercial or financial relationships that could be construed as a potential conflict of interest.
