# Supplementary material for: Genetics of aging, health, and survival: dynamic regulation of human longevity related traits
Source: Front Genet. 2015 Apr 13;6:122. doi: 10.3389/fgene.2015.00122 (PMC4394697; doi:10.3389/fgene.2015.00122)
Supplement: Supplementary file 1 [file Presentation1.PDF]

Supplementary Materials

S.1. Data and methods

In the discovery phase of the analyses we used the EMMAX software to perform GWAS of female lifespan data from the original Framingham Heart Study (FHS) cohorts (Giroux, 2013) as well as genetic data. The analyses used the modified procedure for correction for population stratification described below. The genetic data were represented by 550,000 SNPs. Genotyping was conducted using Affymetrix 500K and 50K (non-overlapping) arrays. The lifespan data were available for 1,529 participants from the original FHS cohort. The quality control (QC) procedure included 95% call rate for the sample and 95% call rate for SNPs, and HWE p-value >1E-7. After applying the QC procedure, the data on 1,111 individuals with information about lifespan and 429,783 SNPs were available for the analysis. Life spans for 204 study subjects (52 males and 152 females) were censored.

Life span imputation. The speed of calculations in GWAS depends on the model used in the statistical association procedure and on how efficiently this procedure was implemented in the corresponding computer program. The relatively fast software used in the genome wide association studies (GWAS) of complex traits that is able to deal with data on related individuals (e.g., family members) is based on a mixed-effect model (e.g., EMMAX software (Kang et al., 2010)). To use such a program in GWAS of human life span, the data for individuals with censored life spans have to be imputed. To make such imputation for a person censored at age x, we calculated the mean residual life span $t(x)$ using data on individuals from the original FHS cohort who survived to age x and died after this age. The imputed life span $T(x)$ for a person censored at age x was obtained using formula:

$$T(x) = x + t(x)$$

This procedure was performed for females and males. As a result we obtained complete life span data sets on 679 females and 432 males.

Modified procedure for controlling for potential population stratification. An important problem for GWAS of complex traits is how to deal with possible population stratification due to differences in ancestry. Being uncontrolled, such stratification may bias the results of analyses, leading to false-positive findings and misleading conclusions. A procedure widely used in controlling for possible effects of population stratification in GWAS of complex traits is based on principal component analysis (PCA) of genetic data (Price et al., 2006). The method identifies several top principal components (PCs) and uses them as observed covariates in the association analyses.

For many complex traits the use of such procedure keeps researchers out of trouble. However, in the genetic studies of human longevity this approach has to be used with care. This is because the process of mortality selection in genetically heterogeneous populations may generate additional genetic structure in the study population. This additional structure which involves genetic variants affecting life span may be inadvertently captured by the selected principal components. If that happens, then controlling for the potential effects of population stratification in GWAS of human longevity related traits may substantially reduce the estimates of associations of genetic variants with these traits, i.e., it may weaken the signals from the genetic variants one is trying to detect. This situation indicates possible cause of weak genetic signals observed in many recent GWAS of human aging and longevity and provides useful insight into how this problem could be addressed. To increase the strength of genetic signals one has to separate the two sources causing population genetic

structure and construct PCs that capture only the source of population stratification due to differences in ancestry. Ideally, the data on the members of the original FHS cohort below 60 years could be used for this purpose. The 60-years limit is taken because up to this age mortality selection process was not able to modify genetic structure using genes we are interesting in. However, the number of such individuals is small and therefore cannot be used for constructing such PCs. An alternative solution is to use the fact that differences in ancestry among individuals in the original FHS cohort are also represented in the genetic structure of their children comprising the offspring FHS cohort. Following this idea we selected independent individuals from the offspring FHS cohort below age 60 years at the time of bio-specimen collection and used them for constructing PCs. Altogether 1,625 such individuals were selected. Then following the procedure described in Price et al (2006) we assigned values of PC to the members of the original FHS and offspring cohort. The idea of this approach to controlling for population stratification, the results of analyses, as well as comparison of the results of traditional and improved genetic analyses of data on human life span are summarized in Yashin et al. (2014).

Discovery phase. We applied a mixed-effects model using the EMMAX software (Kang et al., 2010) to perform GWAS of human life span for 679 females from the original FHS cohort using the first 20 PCs, smoking habit (ever or never), and birth cohort as observed covariates. In these analyses the additive genetic model of the connection between genetic variants and life span was used.

Confirmation phase. In the confirmation phase we used genetic and the life span data on males from the same (original) FHS cohort and the life span data on males and females combined from the offspring FHS cohort.

Testing for pleiotropic effects. Using available data on cancer (all sites but skin) and CVD as well as longitudinal data on physiological variables we estimated age trajectories of probabilities of staying free of corresponding diseases for carriers and non-carriers of selected genetic variants as well as association of these variants with age trajectories of physiological indices. We also investigated how smoking modulates estimated associations. We used data on ages at disease onset (cancer of all sites but skin, CVD) from the same cohort to evaluate genetic effects on incidence rates of these health disorders. The occurrence of cardiovascular diseases (CVD), cancer, and death was identified in the study through continuous surveillance of hospital admissions, death registries, and other sources, so that all events are included in the study. The incidence of cancer was calculated from the dataset "Cancer Cases Reviewed through December 2010". The incidence of CVD was calculated using the "Sequence of Cardiovascular Events" (SOE) files. Individuals who did not contract CVD or cancer during the study were considered censored at the age of death or at the age of the last follow-up. Participants who had CVD/cancer before the first exam were excluded from analysis of joint effects of detected variants on incidence rates of the corresponding health disorder. We used data on smoking habit (ever or never) to evaluate the effects of interactions between selected variants and smoking habit on survival and other traits of interest.

We used longitudinal data on physiological variables to evaluate effects of differences in genetic background, as well as effects of interactions of detected genetic variants with smoking, on aging-related changes developing during the life course. We evaluated the average age trajectories of ten physiological variables: blood glucose (BG); body mass index (BMI); diastolic blood pressure (DBP); hematocrit (HC); systolic blood pressure (SBP); pulse pressure (PP); total cholesterol (CH); ventricular rate (VR); and weight and height. Then we created 13 age groups (<35, 35-39, …., 85-89, and 90+ years) and calculated empirical estimates of the mean values of those physiological variables

in each group using pooled data on measurements from all exams.

Evaluating joint effects of detected genetic variants on survival and other traits. To evaluate joint effects of detected genetic variants on survival we first constructed a polygenic score index by counting the number of detected vulnerability variants carried by each genotyped individual (Yashin et al., 2012). We selected all genetic variants with p-values < 1E-5 for females and males separately. We found 25 SNPs with minor allele having negative effect on survival for females: rs10845099, rs11574358, rs12678797, rs12949468, rs13254175, rs16941236, rs17047321, rs17067605, rs17119093, rs2224842, rs2229188, rs2292664, rs2475028, rs383306, rs4132322, rs41387348, rs4608641, rs4639950, rs4904670, rs5491, rs6983332, rs6986911, rs7689329, rs7894051, rs9628201 and 22 SNPs with minor allele having negative effect on survival for males: rs10845099, rs10914648, rs11574358, rs12435343, rs12949468, rs17067605, rs17121638, rs17836431, rs1794108, rs2229188, rs2292664, rs2586484, rs4639950, rs4886298, rs4904670, rs631503, rs729795, rs7835585, rs7894051, rs902870, rs9628201, rs9928967.

rs_num	*Beta*	*p*	*MAF*	*Chr*	*Gene*	*Function*
rs12949468	-9.225647	5.45E-20	0.1568	17	TLK2	intronic
rs4639950	-7.618965	1.71E-16	0.1455	11	C1QTNF5	5utr
rs217237	9.03618	3.36E-12	0.026	11		
rs7894051	-3.52465	2.52E-10	0.2031	10	ECHS1	intronic
rs4904670	-5.025773	1.50E-09	0.1298	14	NRDE2 (C14orf102)	intronic
rs5491	-4.970503	4.17E-09	0.0942	19	ICAM1	coding
rs3006879	8.20326	4.59E-09	0.02029	1	GLIS1	intronic
rs2292664	-5.827558	1.48E-08	0.08057	12	RIMBP2	coding
rs2229188	-5.445164	1.63E-08	0.08696	7	CYP51A1	coding
rs11574358	5.583841	5.87E-08	0.07407	8	WRN	coding

Table S1. Ten SNPs resulted from GWAS on human life span using female data from the original FHS cohort. The columns include: rs-number, effect size, p-value, minor allele frequencies, chromosome number, gene's name and function.

rs_num	*Beta*	*p*	*MAF*	*Chr*	*Gene*	*Function*
rs12949468	-9.949415	2.68E-17	0.2173	17	TLK2	intronic
rs4639950	-7.7037	1.29E-11	0.2059	11	C1QTNF5	5utr

rs4904670	-6.685775	8.67E-09	0.1976	14	NRDE2 (C14orf102)	intronic
rs7894051	-4.411856	2.87E-07	0.2539	10	ECHS1	intronic
rs2292664	-6.437432	8.46E-07	0.09722	12	RIMBP2	coding
rs2229188	-6.035298	1.55E-06	0.09962	7	CYP51A1	coding
rs11574358	-5.91735	2.58E-06	0.08527	8	WRN	coding
rs5491	-4.288137	1.96E-05	0.1484	19	ICAM1	coding

Table S2. Eight SNPs resulted from genetic analysis of the ten selected SNPs (Table S1) using lifespan data on 432 males from the original FHS cohort. The columns include: rs-number, effect size, p-value, minor allele frequencies, chromosome number, gene's name and function.

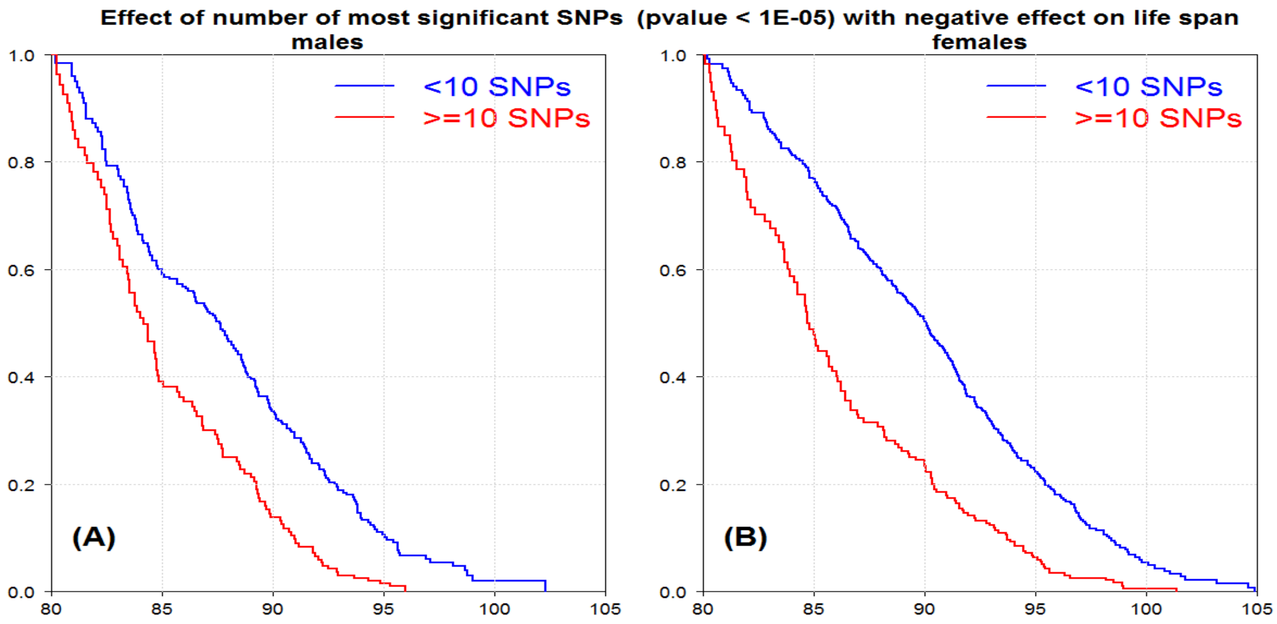

Figure S1. Kaplan Meier estimates of conditional survival functions for groups of individuals surviving to age 80 years and carrying different numbers of vulnerability alleles from the polygenic risks scores constructed from genetic variants showing negative influence on life span with p-value <1.0E-05, for males (A) and females (B). Source: Framingham Heart Study, original cohort, genotyped individuals after quality control.

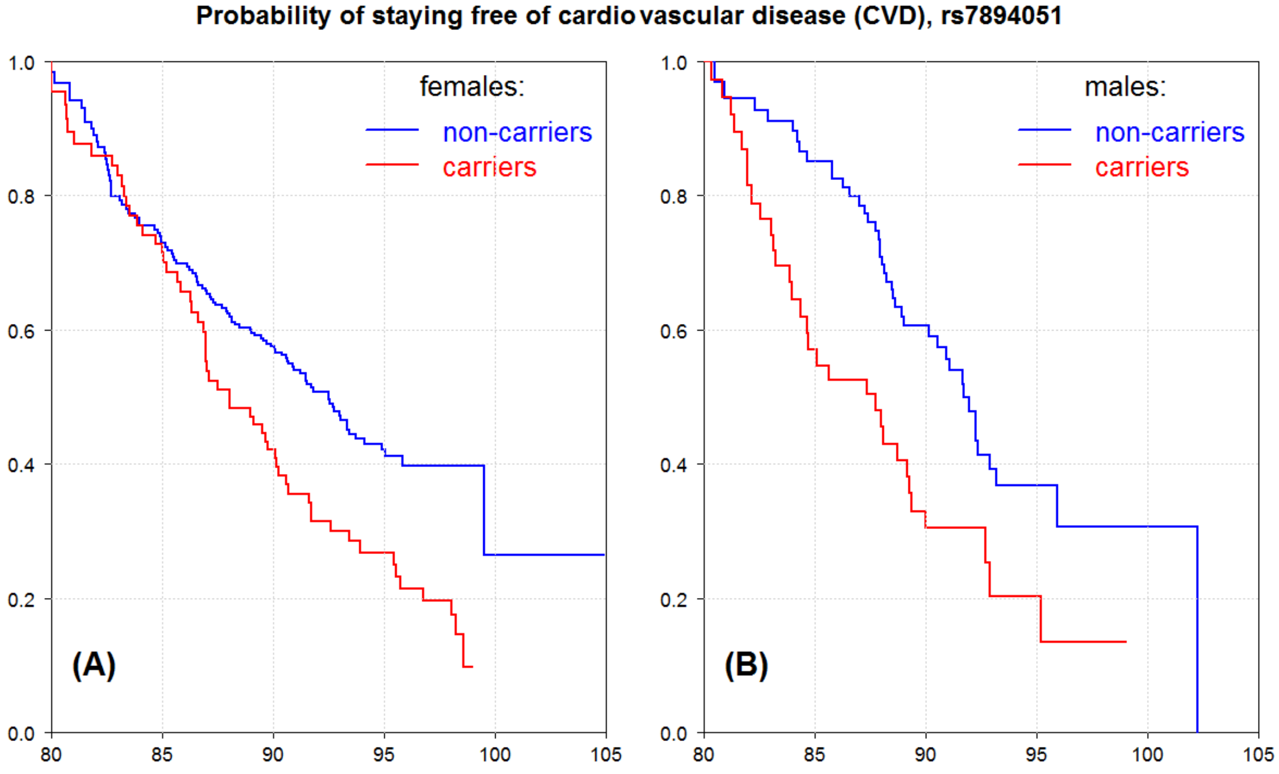

Figure S2. Kaplan-Meier estimates of conditional of probability of staying free of CVD for carriers/non-carriers of minor alleles of rs7894051survived age 80 years for females (A) and males (B). Source: Framingham Heart Study, original cohort, genotyped individuals after quality control.

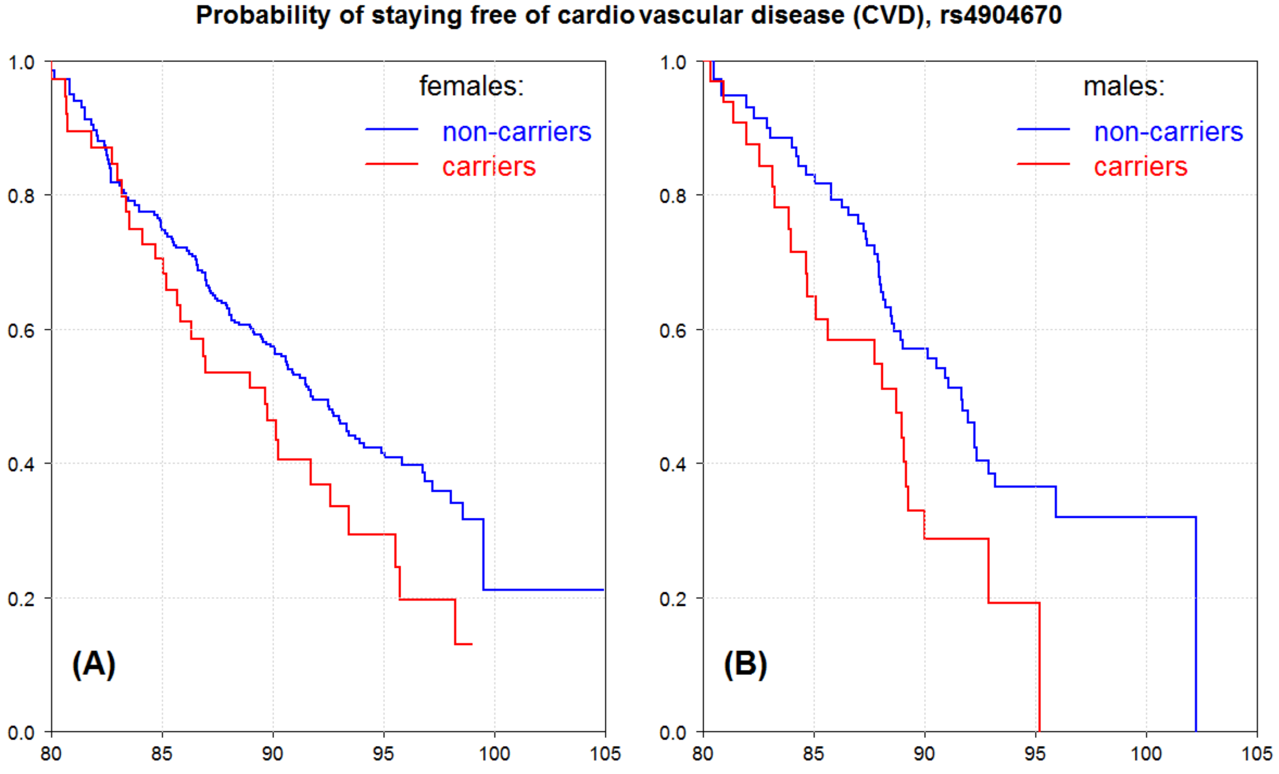

Figure S3. Kaplan-Meier estimates of conditional probability of staying free of CVD for carriers/non-carriers of minor alleles of rs4904670 surviving to age 80 years for females (A) and males (B). Source: Framingham Heart Study, original cohort, genotyped individuals after quality control.

SNP name	*N_T*	*N_E*	*Beta*	*SE*	*Pvalue*	*HR*	*HRlow*	*HRup*
rs5491	928	724	0.662	0.093	1.13E-12	1.938	1.615	2.326
rs2229188	960	756	0.617	0.094	5.74E-11	1.854	1.541	2.230
rs11574358	952	748	0.601	0.101	2.41E-09	1.824	1.497	2.221
rs7894051	933	730	0.439	0.068	9.36E-11	1.551	1.358	1.771
rs4639950	943	741	0.617	0.081	3.17E-14	1.853	1.580	2.172
rs2292664	926	733	0.620	0.098	2.50E-10	1.859	1.534	2.252
rs4904670	924	721	0.590	0.084	1.70E-12	1.804	1.531	2.125
rs12949468	960	756	0.611	0.079	1.50E-14	1.842	1.576	2.153

Table S3. Effect of individual genetic variants on all-cause mortality risk for individuals surviving to age 80 in the FHS original cohort estimated using Cox proportional hazards model. HRlow and HRup denote 95% interval for hazard ratio (HR)

SNP name	*N_T*	*N_E*	*Beta*	*SE*	*Pvalue*	*HR*	*HRlow*	*HRup*
rs5491	928	187	0.81	0.18	4.2E-06	2.24	1.59	3.15
rs2229188	960	194	0.71	0.18	8.0E-05	2.04	1.43	2.90
rs11574358	952	190	0.63	0.20	1.5E-03	1.88	1.27	2.76
rs7894051	933	185	0.47	0.13	4.3E-04	1.59	1.23	2.07
rs4639950	943	187	0.70	0.16	9.3E-06	2.02	1.48	2.75
rs2292664	926	189	0.68	0.19	3.0E-04	1.98	1.37	2.86
rs4904670	924	185	0.60	0.16	2.2E-04	1.83	1.33	2.52
rs12949468	960	194	0.69	0.15	8.5E-06	1.99	1.47	2.69

Table S4. Effect of individual genetic variants on risk of mortality from CVD for individuals surviving to age 80 in the FHS original cohort estimated using Cox proportional hazards model. HRlow and HRup denote 95% interval for hazard ratio (HR); N_T denotes the total number of individuals; N_E is the total number of events

SNP name	*N_T*	*N_E*	*Beta*	*SE*	*Pvalue*	*HR*	*HRlow*	*HRup*
rs5491	928	115	0.585	0.226	0.010	1.796	1.152	2.798
rs2229188	960	120	0.548	0.237	0.021	1.730	1.087	2.753
rs11574358	952	119	0.475	0.257	0.065	1.608	0.971	2.663
rs7894051	933	117	0.362	0.169	0.032	1.437	1.031	2.002
rs4639950	943	117	0.496	0.204	0.015	1.642	1.100	2.452
rs2292664	926	116	0.530	0.247	0.032	1.699	1.046	2.757
rs4904670	924	114	0.506	0.209	0.015	1.659	1.103	2.497
rs12949468	960	120	0.496	0.199	0.013	1.642	1.112	2.426

Table S5. Effect of individual genetic variants on risk of mortality from cancer for individuals

surviving to age 80 years in the FHS original cohort estimated using Cox proportional hazards model. HRlow and HRup denote 95% interval for hazard ratio (HR); N_T denotes the total number of individuals; N_E is the total number of events.

SNP name	*N_T*	*N_E*	*Beta*	*SE*	*Pvalue*	*HR*	*HRlow*	*HRup*
rs5491	535	210	0.335	0.189	0.07556	1.398	0.966	2.023
rs2229188	557	220	0.503	0.182	0.00561	1.654	1.159	2.361
rs11574358	554	217	0.475	0.196	0.01522	1.608	1.096	2.359
rs7894051	545	216	0.452	0.125	0.00031	1.572	1.230	2.010
rs4639950	547	213	0.466	0.158	0.00323	1.594	1.169	2.175
rs2292664	539	216	0.511	0.188	0.00668	1.667	1.152	2.412
rs4904670	535	210	0.423	0.161	0.00838	1.527	1.115	2.092
rs12949468	557	219	0.476	0.153	0.00186	1.609	1.193	2.171

Table S6. Effect of individual genetic variants on risk of onset of CVD for individuals surviving to age 80 in the FHS original cohort estimated using Cox proportional hazards model. HRlow and HRup denote 95% interval for hazard ratio (HR); N_T denotes the total number of individuals; N_E is the total number of events.

SNP name	*N_T*	*N_E*	*Beta*	*SE*	*Pvalue*	*HR*	*HRlow*	*HRup*
rs5491	1089	264	1.389	0.198	2.4E-12	4.011	2.720	5.914
rs2229188	1092	266	0.869	0.219	7.1E-05	2.385	1.553	3.663
rs11574358	1088	264	0.935	0.240	9.7E-05	2.547	1.592	4.076
rs7894051	1086	265	0.538	0.160	0.00079	1.713	1.251	2.344
rs4639950	1079	263	1.144	0.208	3.9E-08	3.138	2.087	4.719
rs2292664	1027	249	1.388	0.235	3.3E-09	4.008	2.531	6.348
rs4904670	1084	263	0.984	0.206	1.8E-06	2.676	1.787	4.007
rs12949468	1090	265	1.062	0.201	1.3E-07	2.893	1.950	4.291

Table S7. Effect of individual genetic variants on all-cause mortality risk conditional on survival to age 75 in the FHS offspring cohort estimated using Cox proportional hazards model. HRlow and HRup denote 95% interval for hazard ratio (HR); N_T denotes the total number of individuals; N_E is the total number of events

S.2. The quality of genetic data

The quality of genetic data is a matter of serious concern in GWAS of complex traits. This issue is especially important when genetic data on members of the original FHS cohort are used. This dataset was one of the first prepared for genetic analyses using GWAS, and a substantial portion of genotyped cohort members died before the quality of genotyping was substantially improved. As a consequence, these data have higher chances of having genotyping errors than more recently produced datasets. Taking this into account, we performed testing of the quality of genotyping for eight genetic variants detected in this study using special files distributed by Affymetrix that are included in the FHS dataset provided by the dbGaP. The file dealing with 500K SNP set contained two SNPs from this list. Both got the “PASS” mark in the “Recommended Status” column of this file. The other four files contain the quality control metrics estimated under different values of the call rate for SNPs from the 50K SNP set. According to the “Phase 1-genomic DNA” file all remaining six SNPs selected in our study got the mark “PASS” in the Recommended Status column

of this file. In the “Phase 2-genomic DNA” file only five out of six SNPs were marked “PASS” in the Recommended Status column. The rs2229188 was marked “FAIL” in this column. In the two remaining Affymetrix files where the call rates were relaxed, all six SNPs were marked “FAIL” in the Recommended Status columns. These results confirmed the importance of using stringent requirements for the control rates in genetic analyses.

Note that although the Affymetrix files inform users that subsequent files used lower call rates, they did not specify the particular levels of these call rates. Taking this into account, we performed additional quality control testing for eight genetic variants with QC specification used in our study (95% call rate for sample, the Hardy-Weinberg Equilibrium (HWE) p-value > 1.0E-7). We tested the HWE for SNP loci of detected variants by applying PLINK software to the data on female members of the original FHS because our analyses started by using these data. PLINK has two options for calculating the HWE *p*-value. One uses only data on the population of founders (i.e., unrelated individuals from this cohort). The other option uses the population of all individuals (founders and non-founders) available in this cohort. We found that under the “founders” PLINK option all detected SNPs had HWE *p*-values > 5.2E-06. The use of the “founders and non-founders” option resulted in HWE *p*-values > 2.64E-07 for all eight detected SNPs. Both these estimates are within the limits specified in the QC procedure used in our analyses.

Repeating this procedure for males of the same cohort, we found HWE *p*-values > 2.1E-07 for all eight detected SNPs under the “founders” option. The use of “founders and non-founders” option resulted in HWE *p*-values of 6.8E-09 for rs4639950 and 2.9E-10 for rs12949468. For remaining six SNPs, the HWE *p*-values were > 2.9E-07. The HWE *p*-value threshold used in QC specification in our study corresponded to the “founders” option in PLINK.

The application of this procedure to the data on all three FHS cohorts under the PLINK “founders and non-founders” option dramatically reduced the HWE *p*-value for the rs2229188 SNP but kept the values for other SNPs larger than the 1.0E-7 limit. These results were observed for males, females, and males and females combined and indicate possible genotyping error in the rs2229188. The use of the PLINK “founders” option resulted in HWE *p*-values > 1.0E-07 for all selected SNPs (including rs2229188).

The presence of all three generations of study participants in the FHS data created an opportunity for testing the presence of Mendelian inheritance error by comparing offspring genotypes with those of their parents in the original FHS cohort. Large values of this error indicate high chances of genotyping error. The 2% level is considered acceptable for such errors (the estimates of Mendelian inheritance error in SNPs that got the mark “Pass” in the Affymetrix files did not exceed 2%). We calculated the Mendelian inheritance error using genetic data on families that included members of the original and offspring FHS cohorts. The errors were relatively small for all eight SNPs. The error increased substantially for the rs2229188 SNP when all three FHS generations were included in the calculations. These results provided additional evidence on possible genotyping error in the rs2229188 SNP. Note that indications of such error appeared each time when data on subsequent FHS generations were included in the analysis. We also found higher levels of minor allele frequencies of detected SNPs in the original FHS cohort compared to those in the offspring FHS cohort. These differences, however, may depend on many additional factors (e.g., assortative mating). Finally, we also investigated SNP scatter plots for each of eight selected SNPs. As a result of these analyses we selected two SNPs rs7894051 (in ECHS1 gene), and rs4904670 (in NRDE2 gene) for further analyses of their associations with major diseases (cancer and CHD) and physiological aging changes.

The results of this QC testing indicated that although detected genetic associations were confirmed twice (findings on female data were confirmed using data on males from the original FHS cohort,

and then using data from the offspring FHS cohort), additional studies with high quality genetic data are needed to make final conclusions about the involvement of the detected genes and corresponding pathways in regulation of health and longevity traits.

References

Giroux, E. (2013). The Framingham Study and the Constitution of a Restrictive Concept of Risk Factor. *Social History of Medicine* 26**,** 94-112. doi: 10.1093/shm/hks051.

Kang, H.M., Sul, J.H., Service, S.K., Zaitlen, N.A., Kong, S.-Y., Freimer, N.B., Sabatti, C., and Eskin, E. (2010). Variance component model to account for sample structure in genome-wide association studies. *Nature Genetics* 42**,** 348-354. doi: 10.1038/ng.548.

Price, A.L., Patterson, N.J., Plenge, R.M., Weinblatt, M.E., Shadick, N.A., and Reich, D. (2006). Principal components analysis corrects for stratification in genome-wide association studies. *Nature Genetics* 38**,** 904-909. doi: 10.1038/ng1847.

Yashin, A.I., Wu, D., Arbeev, K.G., Arbeeva, L.S., Akushevich, I., Kulminski, A., Culminskaya, I., Stallard, E., and Ukraintseva, S.V. (2014). Genetic structures of population cohorts change with increasing age: Implications for genetic analyses of human aging and life span. *Annals of Gerontology and Geriatric Research* 1**,** 1020.

Yashin, A.I., Wu, D., Arbeev, K.G., Stallard, E., Land, K.C., and Ukraintseva, S.V. (2012). How genes influence life span: the biodemography of human survival. *Rejuvenation Res* 15**,** 374-380. doi: 10.1089/rej.2011.1290.
